# FGF19 protects skeletal muscle against obesity‐induced muscle atrophy, metabolic derangement and abnormal irisin levels via the AMPK/SIRT‐1/PGC‐α pathway

**DOI:** 10.1111/jcmm.16448

**Published:** 2021-03-10

**Authors:** Ai Guo, Kai Li, Hong‐Chuan Tian, Zhen Fan, Qiu‐Nan Chen, Yun‐Fei Yang, Jing Yu, Yong‐Xin Wu, Qian Xiao

**Affiliations:** ^1^ Department of Geriatrics The First Affiliated Hospital of Chongqing Medical University Chongqing China; ^2^ Department of Orthopedics The First Affiliated Hospital of Chongqing Medical University Chongqing China; ^3^ Department of Orthopedics The Second Affiliated Hospital of Chongqing Medical University Chongqing China

**Keywords:** FGF19, insulin resistance, irisin, lipid accumulation, muscle atrophy, obesity, sarcopenic obesity

## Abstract

Obesity is associated with biological dysfunction in skeletal muscle. As a condition of obesity accompanied by muscle mass loss and physical dysfunction, sarcopenic obesity (SO) has become a novel public health problem. Human fibroblast growth factor 19 (FGF19) plays a therapeutic role in metabolic diseases. However, the protective effects of FGF19 on skeletal muscle in obesity and SO are still not completely understood. Our results showed that FGF19 administration improved muscle loss and grip strength in young and aged mice fed a high‐fat diet (HFD). Increases in muscle atrophy markers (FOXO‐3, Atrogin‐1, MuRF‐1) were abrogated by FGF19 in palmitic acid (PA)‐treated C2C12 myotubes and in the skeletal muscle of HFD‐fed mice. FGF19 not only reduced HFD‐induced body weight gain, excessive lipid accumulation and hyperlipidaemia but also promoted energy expenditure (PGC‐1α, UCP‐1, PPAR‐γ) in brown adipose tissue (BAT). FGF19 treatment restored PA‐ and HFD‐induced hyperglycaemia, impaired glucose tolerance and insulin resistance (IRS‐1, GLUT‐4) and mitigated the PA‐ and HFD‐induced decrease in FNDC‐5/irisin expression. However, these beneficial effects of FGF19 on skeletal muscle were abolished by inhibiting AMPK, SIRT‐1 and PGC‐1α expression. Taken together, this study suggests that FGF19 protects skeletal muscle against obesity‐induced muscle atrophy, metabolic derangement and abnormal irisin secretion partially through the AMPK/SIRT‐1/PGC‐α signalling pathway, which might be a potential therapeutic target for obesity and SO.

## 1 INTRODUCTION

Obesity has become an unprecedented individual and social medical challenge that increases the risk of metabolic diseases, including hypertension, dyslipidaemia and type 2 diabetes mellitus (T2D).^1^ Sarcopenic obesity (SO) is the coexistence of obesity and sarcopenia (age‐related muscle loss and physical dysfunction). SO has become a major health burden in the ageing population because of the increasing prevalence and serious consequences.^2^ SO is considered to cause a much higher risk of morbidity and mortality compared with obesity or sarcopenia alone.^3^ Ectopic fat deposition induced by obesity induces biological dysfunctions of skeletal muscle including insulin resistance (IR), mitochondrial dysfunction, oxidative stress and inflammation.^4^ These changes further aggravate skeletal muscle loss and physical dysfunction.^5^ The underlying mechanisms of SO are considered to be more complicated than those of obesity or sarcopenia alone.^6^ The current treatment for SO mainly focuses on lifestyle interventions and pharmacologic therapies.^7^ However, some controversies and limitations still persist in the diagnosis and treatment of SO.^8^ Therefore, the identification of therapeutic targets for SO has gained a lot of attention.

Skeletal muscle is well known to produce and secrete various myokines to mediate muscle metabolism and maintain body homeostasis.^9^, ^10^ Irisin, as a recently identified myokine, is mainly produced from secreted from skeletal muscle and adipose tissue. This myokine is secreted into the circulation after the proteolytic cleavage of fibronectin type III domain‐containing protein 5 (FNDC‐5).^11^ Studies have shown that peroxisome proliferator‐activated receptor γ coactivator 1α (PGC‐1α) is involved in regulating FNDC‐5 production.^12^ Irisin not only promotes the browning of white adipose tissue (WAT) but also regulates the biological activities of skeletal muscle.^13^ This myokine has been identified to regulate the development of obesity, T2D, and other metabolic‐related diseases.^14^

Human fibroblast growth factor 19 (FGF19) is an endocrine hormone that is mostly secreted from ileal enterocytes.^15^ FGF19 has been reported to have the ability to regulate the growth of organs and tissues, bile acid synthesis, fatty acid oxidation, glucose metabolism and mitochondrial functions.^16^, ^17^, ^18^, ^19^ Thus, it has become a pharmacologic target for metabolic diseases, including nonalcoholic fatty liver disease (NALFD), obesity and T2D.^20^, ^21^, ^22^ A recent study found that FGF19 could promote muscle hypertrophy and protect against muscle wasting by activating the extracellular signal‐regulated kinase 1/2 (ERK1/2) and mammalian target of rapamycin (mTOR)‐dependent pathway.^23^ However, the other underlying mechanisms of FGF19 in skeletal muscle are not completely clear.

It is well known that AMP‐activated protein kinase (AMPK) and Sirtuin‐1 (SIRT‐1) act as crucial regulators of biological functions in skeletal muscle, such as muscle atrophy, glucose and lipid metabolism, myokine secretion and mitochondrial function.^24^, ^25^ PGC‐1α, as a downstream transcription regulator mediated by AMPK and SIRT‐1, is associated with abundant biological pathways in skeletal muscle.^26^ Our previous study has suggested that FGF19 can attenuate mitochondrial dysfunction and oxidative stress induced by palmitic acid (PA) through the AMPK/PGC‐1a signalling pathway in C2C12 myotubes.^27^ In this study, we further hypothesized that FGF19 could also ameliorate muscle atrophy, lipid and glucose metabolic derangement and abnormal irisin levels via the AMPK/SIRT‐1/PGC‐1α signalling pathway in PA‐treated myotubes and in the skeletal muscle of HFD‐fed mice.

## 2 MATERIALS AND METHODS

### 2.1 Cell culture and treatment

C2C12 mouse myoblasts (ATCC) were cultured in Dulbecco's Modified Eagle's Medium (DMEM, Gibco) containing 4.5 g/L D‐glucose, 10% foetal bovine serum (Gibco) and 1% penicillin‐streptomycin solution (HyClone) in appropriate conditions. When the cells reached 90%‐100% confluence, they were incubated in DMEM containing 2% horse serum (HS, Bioind, Israel) to promote differentiation into myotubes for 5 days. The myotubes were treated with 100 ng/mL FGF19 (R&D) and 0.5 mM PA (Sigma‐Aldrich) for 24 hours. The dissolution method and concentration selection of FGF19 and PA were consistent with our previous study.^22^

### 2.2 siRNA interference and inhibitor intervention

The PGC‐1α‐siRNA was transfected into C2C12 cells in 6‐well plates using EndoFectin™‐MAX Transfection Reagent (GeneCopoeia) following the manufacturer's protocol. The sequence of PGC‐1α‐siRNA and nontargeting siRNA was as shown: (forward, F): 5′‐UCCAGUAAGCACACGUUUAUU‐3′, (reverse, R): 5′‐AAUAAACGUGUGCUUACUGGA ‐3′; forward, F): 5′‐UUCUCCGAACGUGUCACGUdTdT‐3′, (reverse, R): 5′‐ACGUGACACGUUCGGAGAAdTdT‐3′. Scrambled nontargeting siRNA was used as a negative control. Extents of PGC‐1α knockdown were measured and analysed by RT‐PCR and western blotting. The C2C12 cells were continuously incubated in fresh medium for 24 hours and treated with PA and FGF19 for an additional 24 hours for use in subsequent experiments. In addition, the AMPK and SIRT‐1 inhibitors, Compound C (CC, Dorsomorphin, Med Chem Express) and EX‐527 (Med Chem Express) were used to suppress AMPK and SIRT‐1 expression. The differentiated C2C12 cells were treated with FGF19 and PA in the presence or absence of CC and EX‐527 for 24 hours.

### 2.3 Enzyme‐linked immunosorbent assay (ELISA)

Irisin concentrations in the cell culture supernatant were identified using enzyme‐linked immunosorbent assay (ELISA) kits (J&L Biologica) following the manufacturer's directions.

### 2.4 Immunofluorescence

The C2C12 myotubes were fixed with 4% paraformaldehyde (PFA) for 30 minutes and permeabilized with 0.3% Triton‐X for 15 minutes. After washing with PBS, the cells were blocked with 10% normal goat serum for 1 hour and incubated with anti‐myosin heavy chain (MHC) antibody (1:200, Santa Cruz) overnight at 4°C. After washing, the cells were incubated with Alexa Fluor 488‐conjugated goat anti‐mouse IgG antibody (ZSGB‐BIO, China) for 1 hour. The nuclei were stained with DAPI (Boster). The fusion index was calculated as the percentage of the number of nuclei in MHC‐positive myotubes (>2 nuclei) to the total number of nuclei. Images were obtained by laser confocal scanning microscopy (LCSM, Zeiss) and analysed using ImageJ software.

### 2.5 Glucose uptake (2‐NBDG)

C2C12 cells were incubated in specialized confocal culture dishes with or without intervention and then treated with Krebs‐Ringer‐Bicarbonate (KRB) buffer (Leagene) containing 100 µM fluorescent deoxyglucose analogue (2‐NBDG, APExBIO) for 30 minutes. After washing with KRB buffer, glucose uptake was evaluated by LSCM.

### 2.6 Oil red O staining

After intervention, the myotubes were washed with PBS twice and then incubated with Oil Red O working solution (Servicebio) for 15 minutes at 37°C. The cells were visualized by light microscopy (Olympus).

### 2.7 Animal experiments

Six‐week‐old and fifteen‐month‐old male C57BL/6J mice were purchased from the Animal Center of Chongqing Medical University (Chongqing, China). Mice were housed in a pathogen‐free environment with a temperature of 22 ± 2°C, humidity of 60% and 12‐hour light/12‐hour dark cycles. After 1 week of adaption, the young and aged mice were randomly fed a standard rodent chow diet (18 kcal% fat, 24 kcal% protein, 58 kcal% carbohydrate; Research Diets, Inc) or an HFD (60 kcal% fat, 20 kcal% protein, 20 kcal% carbohydrate; Research Diets, Inc) for more than 5 months. The mouse models of obesity and sarcopenic obesity were evaluated by body weight gain, fat weight, muscle mass and grip strength. The adult and aged mice were further divided into the following groups, respectively (n = 6 per group): control diet‐fed group (Con), HFD‐fed group (HFD), FGF19‐treated control group (FGF19), and FGF19‐treated HFD‐fed group (FGF19 + HFD). Recombinant FGF19 (0.1 mg/kg, ProSpec CYT‐700, diluted with 0.1% BSA) was administered via intraperitoneal injection daily for 3 consecutive weeks. All animal experiments were conducted in accordance with standard animal care protocols. This study was approved by the Animal Ethics Committee of The First Affiliated Hospital of Chongqing Medical University.

### 2.8 Intraperitoneal glucose tolerance test (IPGTT) and serological assay

After 12 hours of fasting, the mice were subjected to an intraperitoneal injection of 20% glucose solution at a dose of 1.5 g/kg of body weight. The glucose concentration from tail blood was measured at 0, 15, 30, 60, 90 and 120 minutes using a glucometer (Roche Diagnostics GmbH). After all the mice were deeply anaesthetized, blood samples were obtained according to the orbital sinus. Then, all the samples were centrifuged to separate plasma (4°C, 3000 *g*, 10 minutes). The glucose, glycated serum protein (GSP), triglyceride (TG) and total cholesterol (TC) levels were measured using an automatic biochemical analyser (Rayto Chemray 240&800). Circulating FGF19, insulin and irisin levels were also identified by ELISA assay kits (J&L Biologica).

### 2.9 Grip strength test

To verify the physical function of skeletal muscle, the grip strength was determined using an electronic grip strength meter (Cat. 47200, Ugo Basil). After the forelimbs of mice gripped on the sensor lever, their tails were grasped and pulled back until their forepaws released. After three repetitions, the maximal reading was recorded.

### 2.10 Dual‐energy X‐ray absorptiometry (DEXA) test

The mice were placed prone on the bed of the scanner after being anaesthetised with 4% chloral hydrate (0.1 mL/100 g). After scanning, the lean muscle mass was measured by DEXA (Hologic Discovery A, Hologic Inc).

### 2.11 Tissue preparation and histological analysis

Whole gastrocnemius (GAS) muscle (medial and lateral portions), epididymal WAT (eWAT), inguinal WAT (iWAT) and BAT were fixed in 4% PFA. After embedding in paraffin, the samples were cut into 4‐6‐μm‐thick sections. The sections were stained with haematoxylin and eosin (H&E) and Oil Red O solution. The average cross‐sectional area (CSA) of GAS myofibers and the Oil Red O positive area were analysed using Image‐Pro Plus or Image J software.

### 2.12 Immunohistochemistry (IHC)

The GAS muscle sections were deparaffinized in xylene and hydrated in ethanol. After washing with PBS, the sections were incubated in 10% goat serum for 30 minutes at 37°C and then treated with the antibody against FNDC‐5 (1:200, Abcam) overnight at 4°C. The sections were treated with the secondary antibody IgG‐HRP for 30 minutes at 37°C and washed with PBS. Immunostaining was detected by 3,3′ diaminobenzidine tetrahydrochloride (DAB).

### 2.13 Transmission electron microscopy (TEM)

After being cut into 1‐mm^3^ blocks, the GAS muscles were quickly fixed in 2.5% glutaraldehyde. The samples were then further processed for TEM analysis. The images were captured at ×15 000 magnification.

### 2.14 qRT‐PCR

Total RNA was extracted with TRIzol (TaKaRa), and the RNA concentration was determined. cDNA was synthesized with the Prime Script RT reagent kit (TaKaRa). Quantitative real‐time PCR (qRT‐PCR) was performed using the CFX96™ Real‐Time System (Bio‐Rad). The $2^{‐\Delta \Delta C_{t}}$ threshold cycle method was used to analyse the results, and GAPDH was considered as a reference gene. Relative mRNA expression in the control group was normalized to 1. The primers were as follows: PGC‐1α: (F) 5′‐CTGACCACAAACGATGACCCTC‐3′, (reverse, R) 5′‐TGCGGTTGTGTATGGGACTTCT ‐3′; UCP‐1: (F) 5′‐GCTCTTGTTGCCGGGTTTTG‐3′, (R) 5′‐CGTCGGTCCTTCCTTGGTGTA ‐3′; Cidea: (F) 5′‐CTCAATGTCAAAGCCACGATG‐3′, (R) 5′‐ATGTGCCCGCATAGACCAG ‐3′; Cox7a1: (F) 5′‐TAGCTCATCTACCAGAAGCCACT‐3′, (R) 5′‐AGAGTCAGCGTCATGGTCAGTC‐3′; PPAR‐γ: (F) 5′‐CGTGATGGAAGACCACTCGC‐3′, (R) 5′‐TCGCACTTTGGTATTCTTGGAG‐3′; and GAPDH: (F) 5′‐GACATCAAGAAGGTGGTGAAGC‐3′, (R) 5′‐GAAGGTG GAAGAGTGGGAGTT‐3′.

### 2.15 Western blotting

The total protein was separated by SDS‐PAGE and transferred to PVDF membranes. The membranes were incubated with the corresponding primary antibodies, namely anti‐phosphorylated (p)‐AMPK (Thr172; 1:1000; CST), anti‐AMPK (1:1000, CST), anti‐SIRT‐1 (1:1000, Abcam), anti‐p‐FOXO‐3a (Ser253; 1:500; Zen‐Bio Science), anti‐FOXO‐3a (1:1000, GeneTex), anti‐Atrogin‐1 (1:1000, Abcam), anti‐MuRF‐1 (1:1000, Proteintech), anti‐p‐IRS‐1 (Ser307; 1:500; Zen‐Bio Science), anti‐IRS‐1 (1:1000, Zen‐Bio Science), anti‐GLUT‐4 (1:500, Proteintech), anti‐MHC (1:200, Santa Cruz), anti‐MyoD (1:200, Santa Cruz), anti‐Myog (1:200, Santacruz), anti‐PGC‐1α (1:1000, Abcam), anti‐FNDC‐5 (1:1000, Abcam) and anti‐GAPDH (1:5000, Proteintech) overnight at 4°C. Then, the membranes were incubated with the secondary antibodies for 1 h at 37°C. After treatment with an enhanced chemiluminescence substrate kit (Advansta), the protein bands were detected using Fusion software.

### 2.16 Statistical analysis

Data are presented as the mean ± standard deviation (SD). GraphPad Prism 7 was used for the statistical analysis. Differences among groups were determined by one‐way ANOVA followed by Tukey's test for multiple comparisons. The statistically significant threshold was considered as *P* < 0.05.

## 3 RESULTS

### 3.1 FGF19 alleviated PA‐induced muscle atrophy, lipid accumulation, insulin resistance and abnormalities in FNDC5/irisin expression in C2C12 myotubes

**FIGURE 1:**
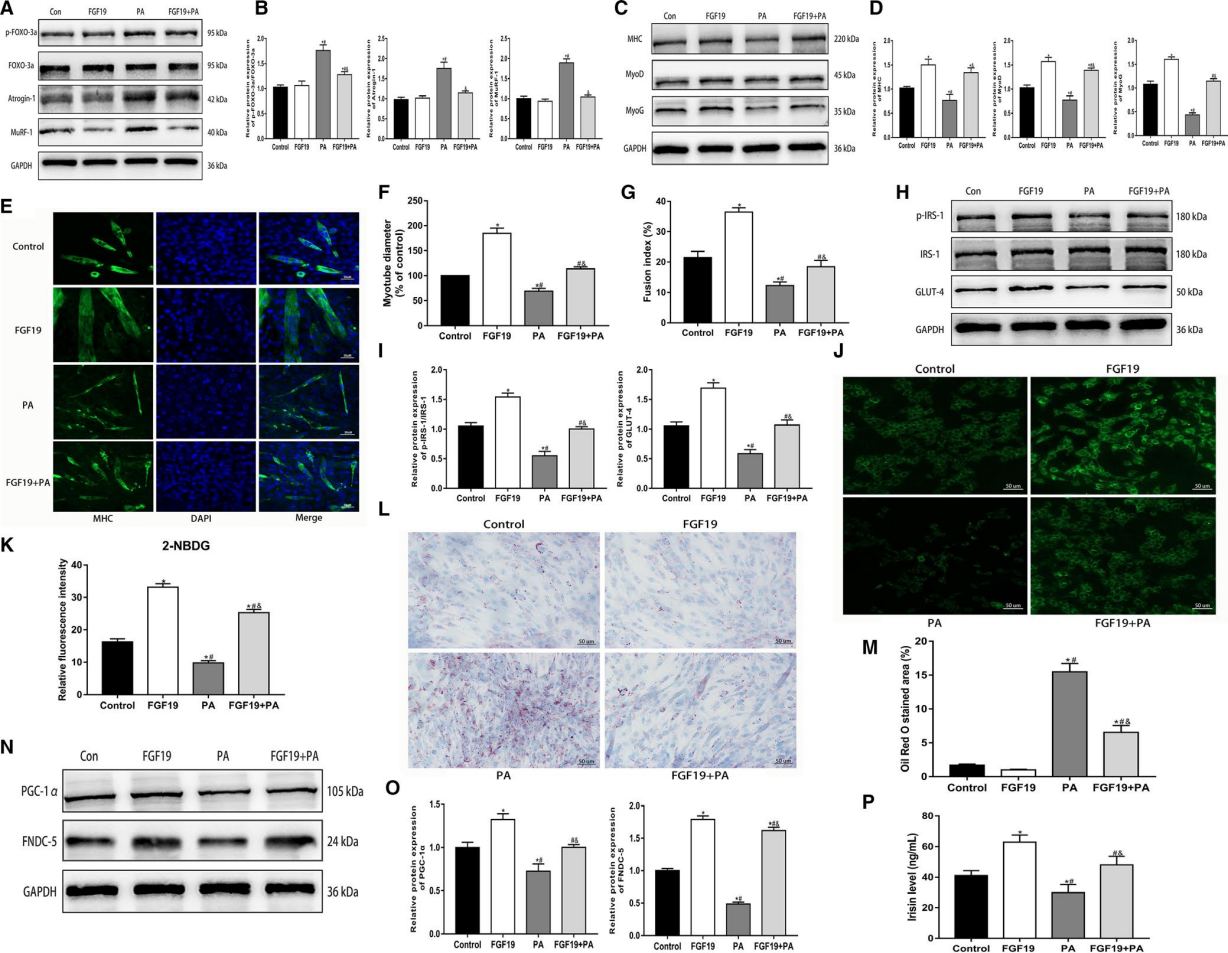
FGF19 alleviated PA‐induced muscle atrophy, lipid accumulation, insulin resistance and abnormalities in FNDC5/irisin expression in C2C12 myotubes. A, B, The protein expression of muscle atrophy markers (p‐FOXO‐3a/FOXO‐3a, Atrogin‐1, and MuRF‐1) was confirmed by western blotting. C, D, The protein expression of myogenic differentiation markers (MHC, MyoD and MyoG) was measured by western blotting. E‐G, Representative images of MHC immunofluorescence (scale bar = 50 μm) in C2C12 myotubes, the diameter of myotubes and the fusion index. H, I, Western blot analysis of p‐IRS‐1/IRS‐1 and GLUT‐4 protein expression. J, K, Representative images of glucose uptake by 2‐NBDG (scale bar = 50 μm). L, M, Representative images of lipid droplet accumulation stained with Oil Red O in C2C12 myotubes (scale bar = 50 μm). N, O, Western blots analysis of PGC‐1α and FNDC‐5 protein expression. P, Irisin levels from cell culture supernatant were identified by ELISA. All data are presented as the mean ± SEM. *P < 0.05 compared with the control group, ^#^ *P* < 0.05 compared with the FGF19 group, ^&^ *P* < 0.05 compared with the PA group

We found that PA significantly increased the protein expression of muscle atrophy markers (p‐FOXO‐3, Atrogin‐1, and MuRF‐1) compared with the control group (Figure 1A,B), while the protein expression of myogenic differentiation markers (MHC, MyoD and MyoG) was dramatically decreased (Figure 1C,D). Furthermore, the immunofluorescence images of MHC showed that PA induced a decrease in myotube diameter and inhibition of the differentiation level by analysis of the fusion index (Figure 1E‐G). However, the PA‐induced aggravation of muscle atrophy and suppression of myotube differentiation could be reversed by FGF19 treatment. These data indicated that FGF19 efficiently alleviated PA‐induced muscle atrophy.

Palmitic acid is widely used to mimic the obese state in vitro, which could induce lipid droplets accumulation and insulin resistance in skeletal muscle. This study found that FGF19 treatment attenuated the PA‐induced reduction in the protein expression of p‐IRS‐1 and GLUT‐4, which is associated with the insulin signalling pathway (Figure 1H,I). Moreover, the glucose uptake test with 2‐NBDG also showed that FGF19 promoted glucose uptake and alleviated PA‐induced insulin resistance to some extent (Figure 1J,K). In addition, Oil Red O staining showed that FGF19 could improve lipid droplet accumulation in PA‐treated myotubes (Figure 1L,M). These results suggested that FGF19 mitigated PA‐induced lipid and glucose metabolic disturbance in C2C12 myotubes.

Our study also found that PA inhibited PGC‐1α and FNDC‐5 expression, resulting in decreased irisin levels in the cell culture supernatant. However, FGF19 restored the PA‐induced reduction in PGC‐1α and FNDC‐5 expression and improved the decreased irisin levels (Figure 1N‐P). These results indicated that FGF19 could attenuate the PA‐induced reduction in irisin levels by enhancing PGC‐1α and FNDC‐5 expression in C2C12 myotubes.

### 3.2 FGF19 attenuated PA‐induced muscle atrophy, glucose and lipid metabolism derangement, and reduced expression of FNDC‐5/irisin via the AMPK pathway

**FIGURE 2:**
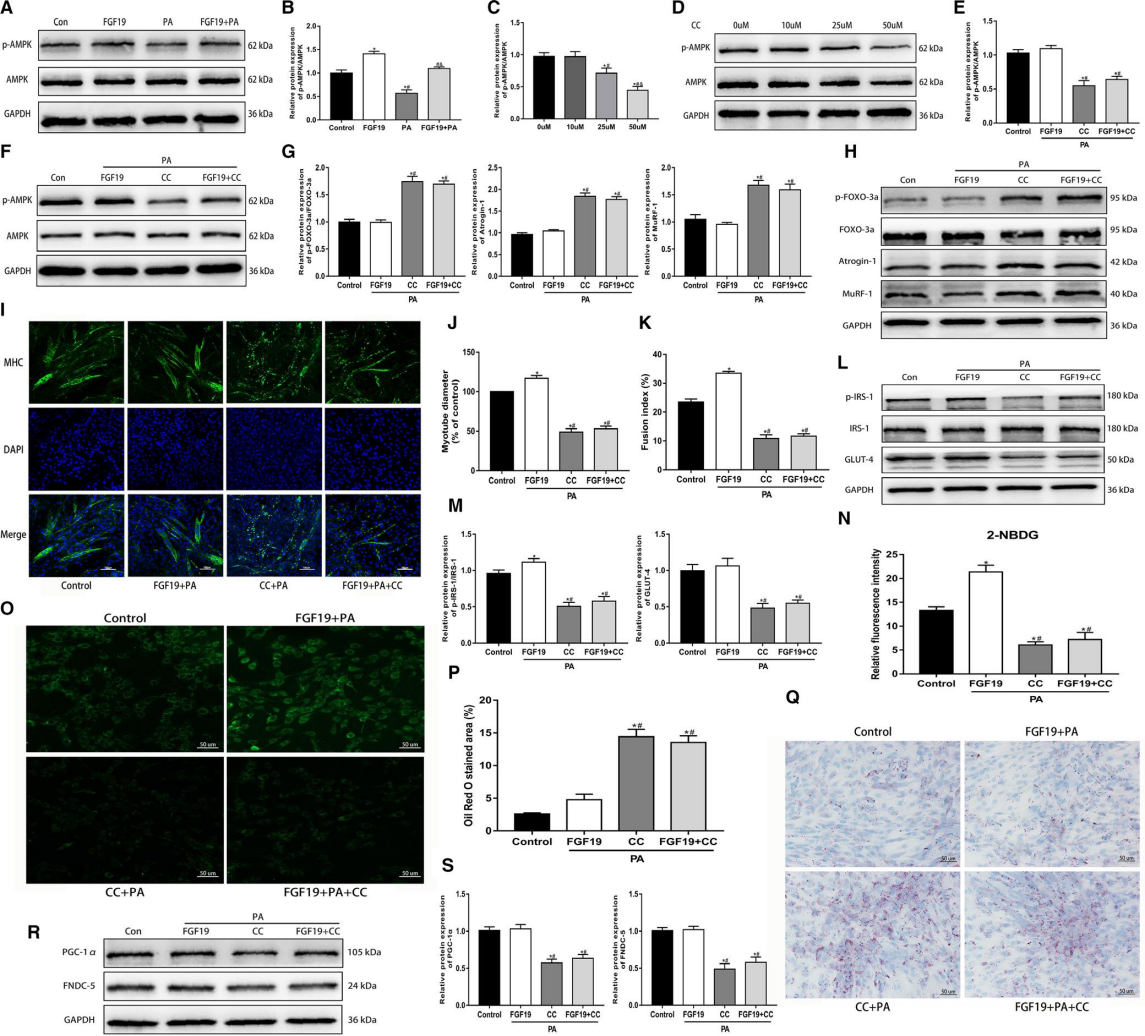
FGF19 attenuated PA‐induced muscle atrophy, glucose and lipid metabolic derangement and reduced FNDC‐5/irisin expression via the AMPK pathway. A, B, Western blot analysis of p‐AMPK/AMPK protein expression in cells treated with FGF19 in the presence or absence of PA. C, D, The effect of different concentrations (0, 10, 25, 50 µM) of the AMPK inhibitor CC on p‐AMPK/AMPK protein expression. E, F, Western blot analysis of p‐AMPK/AMPK protein expression in the control (Con), FGF19 + PA, CC + PA and FGF19 + PA + CC groups. (G‐H) Protein expression of muscle atrophy markers (p‐FOXO‐3a/FOXO‐3a, Atrogin‐1, MuRF‐1) were measured by western blotting. I‐K, Representative images of MHC immunofluorescence (scale bar = 100 μm) in C2C12 myotubes, the diameter of myotubes and the fusion index. L, M, The expression of glucose uptake‐related proteins (p‐IRS/IRS‐1, GLUT‐4) was identified by western blotting. N, O, Representative images of glucose uptake tested by 2‐NBDG (scale bar = 50 μm). P, Q, Representative images of lipid droplet infiltration stained with Oil Red O (scale bar = 50 μm). R, S, Western blot analysis of the expression of irisin production‐related proteins (PGC‐1α, FNDC‐5). All data are presented as the mean ± SEM. *P < 0.05 compared with the control group, ^#^ *P* < 0.05 compared with the FGF19, 10 µM CC or FGF19 + PA group, ^&^ *P* < 0.05 compared with the PA or 25 µM CC group

AMPK plays a critical role in regulating various physiological functions in skeletal muscle, including muscle atrophy, metabolic homeostasis, mitochondrial function and myokine expression. Therefore, this study further verified whether the regulatory effects of FGF19 were related to the activation of AMPK in C2C12 myotubes. First, we found that FGF19 ameliorated PA‐induced decreases in p‐AMPK (Figure 2A,B). Then, we identified the appropriate concentration of AMPK inhibitor, 50 µM CC, for use in the following experiments (Figure 2C,D). We found that the effect of FGF19 on alleviating the PA‐induced decrease in p‐AMPK was abolished by CC intervention (Figure 2E,F).

Moreover, we identified whether AMPK was involved in regulating FGF19‐induced alleviation of PA‐induced muscle atrophy and metabolic derangement and a decrease in irisin expression. This study indicated that CC aggravated the protein expression of muscle atrophy markers and decreased the myotube diameter induced by PA. The improvement of FGF19 for PA‐induced muscle atrophy could be reversed by inhibiting the activation of AMPK (Figure 2G‐K). Immunoblotting showed that compared with the control and FGF19 + PA groups, the protein expression of p‐IRS‐1 and GLUT‐4 was dramatically reduced in the PA + CC and FGF19 + PA + CC groups (Figure 2L,M). Furthermore, the 2‐NBDG test and Oil Red O staining showed that FGF19 did not rescue the PA‐induced impairment in glucose uptake and lipid droplet accumulation with CC intervention (Figure 2N‐Q). In addition, we found that CC inhibited the FGF19‐induced augment in PGC‐1α and FNDC‐5 expression, thus resulting in a decline in irisin production (Figure 2R,S). In conclusion, these results indicated that FGF19 mitigated PA‐induced muscle atrophy, glucose/lipid derangement and the decrease in FNDC‐5/irisin partially through the activation of AMPK.

### 3.3 FGF19 blocked PA‐induced muscle atrophy, glucose and lipid disturbance, and reduced FNDC‐5/irisin expression through the SIRT‐1 pathway

**FIGURE 3:**
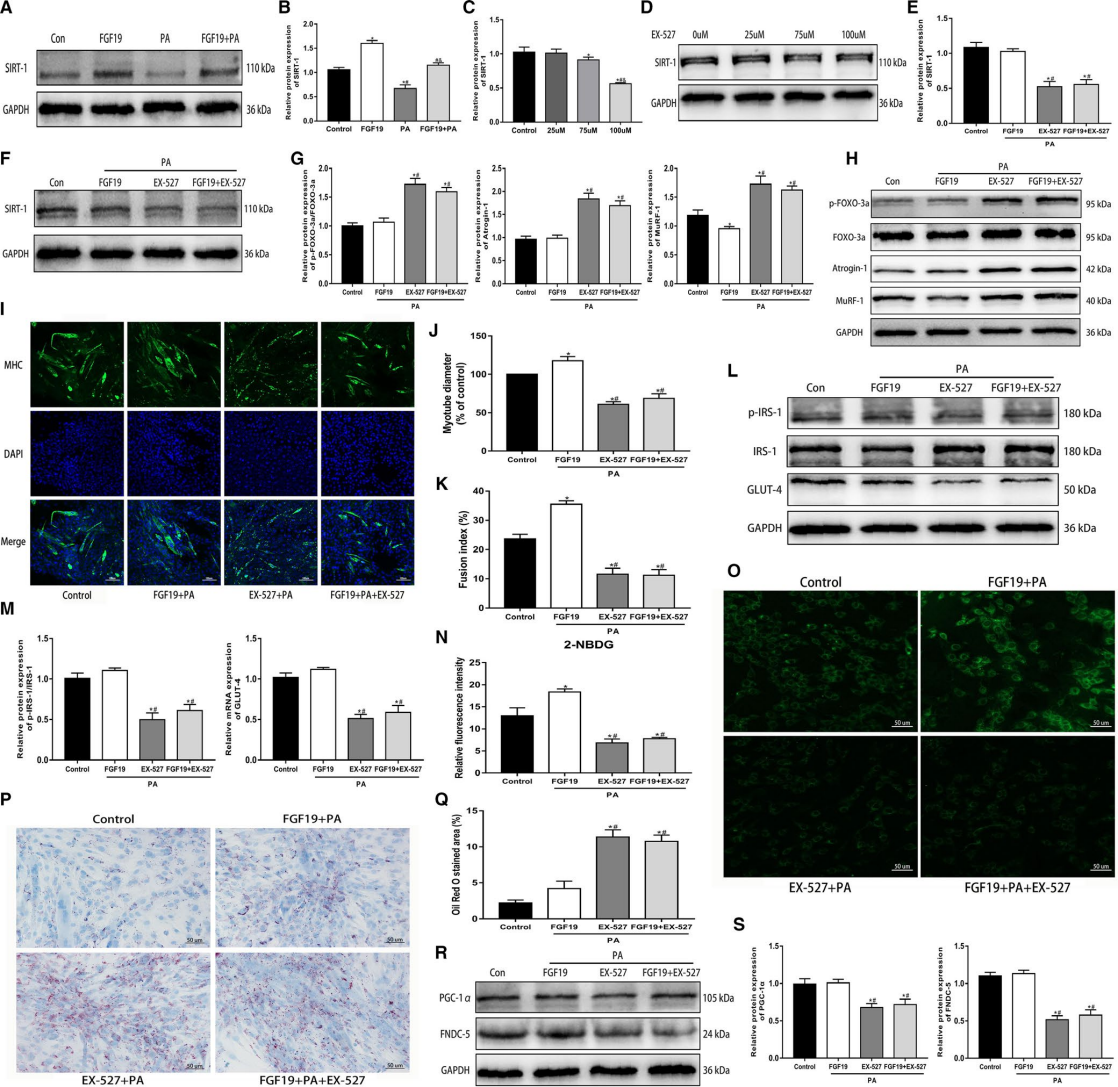
FGF19 blocked PA‐induced muscle atrophy, glucose and lipid disturbance and reduced FNDC‐5/irisin expression through the SIRT‐1 pathway. A, B, Representative western blot images showing SIRT‐1 protein expression in FGF19‐treated C2C12 myotubes in the presence or absence of PA. C, D, The effect of different concentrations (0, 25, 75, 100 µM) of the SIRT‐1 inhibitor EX‐527 on inhibiting SIRT‐1 protein expression was measured by western blotting. E, F, Western blot analysis of SIRT‐1 protein expression in the FGF19 + PA, EX‐527 + PA and FGF19 + PA + EX‐527 groups. G, H, Western blot analysis of the protein expression of muscle atrophy markers (p‐FOXO‐3a/FOXO‐3a, Atrogin‐1, MuRF‐1). I‐K, Representative images of MHC immunofluorescence (scale bar = 100 μm) in C2C12 myotubes, the diameter of myotubes and the fusion index. L, M, Western blot analysis of glucose uptake‐related protein expression (p‐IRS/IRS‐1, GLUT‐4). N, O, Representative images of glucose uptake tested by 2‐NBDG (scale bar = 50 μm). P, Q, Representative images of lipid droplet accumulation stained with Oil Red O (scale bar = 50 μm). R, S, Western blot analysis of the expression of irisin synthesis‐related proteins (PGC‐1α, FNDC‐5). All data are presented as the mean ± SEM. *P < 0.05 compared with the control group, ^#^ *P* < 0.05 compared with the FGF19, 25 µM EX‐527 or FGF19 + PA group, ^&^ *P* < 0.05 compared with the PA or 75 µM EX‐527 group

**FIGURE 4:**
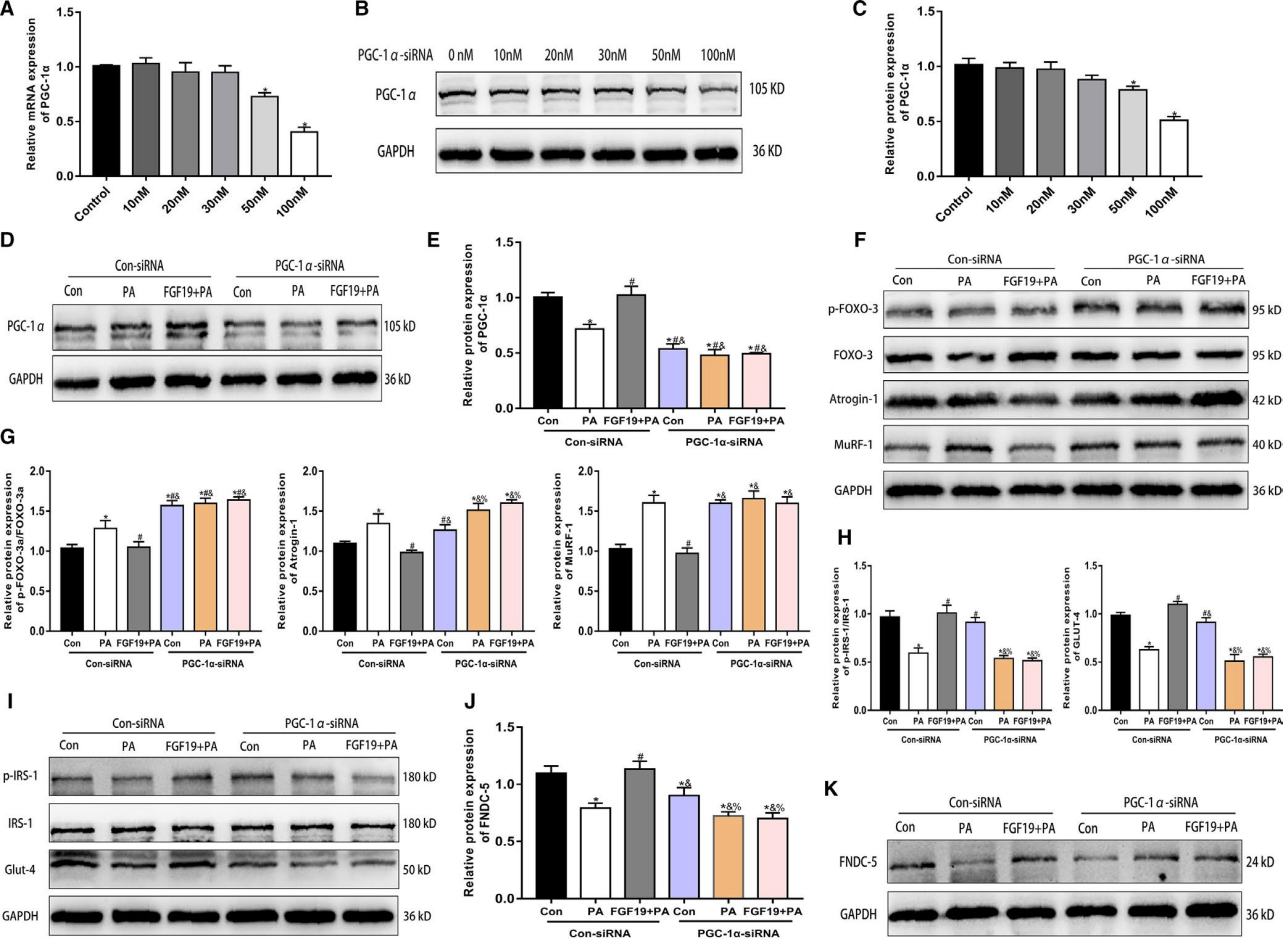
PGC‐1α might be important in mediating the effects of FGF19 on skeletal muscle. The PGC‐1α‐targeted siRNA (PGC‐1α‐siRNA) and scrambled siRNA (Con‐siRNA) were transfected in C2C12 myotubes. A‐C, The transfection efficiency and concentration of PGC‐1α were measured by qRT‐PCR and western blotting. D, E, Representative images and quantitative analysis of PGC‐1α protein expression in PGC‐1α‐siRNA (100 nM) and Con‐siRNA‐transfected C2C12 myotubes treated with PA and FGF19 + PA. F, G, The protein expression of muscle atrophy markers (p‐FOXO‐3a/FOXO‐3a, Atrogin‐1, MuRF‐1) were identified by western blot analysis. H, I, The expression of glucose uptake‐related proteins (p‐IRS/IRS‐1, GLUT‐4) was analysed by western blotting. J, K, Western blot analysis of FNDC‐5 protein expression. All data are presented as the mean ± SEM, *P < 0.05 compared with the control group or the Con‐siRNA group, ^#^ *P* < 0.05 compared with the PA + Con‐siRNA group, ^&^ *P* < 0.05 compared with the FGF19 + PA + Con‐siRNA group, ^%^ *P* < 0.05 compared with the PGC‐1α‐siRNA group

SIRT‐1 is involved in mediating various biological functions in skeletal muscle. We found that compared with the control condition, PA significantly decreased SIRT1 protein expression, and FGF19 could restore the PA‐induced decrease in SIRT1 expression (Figure 3A,B). To verify whether SIRT‐1 was associated with mediating the biological effects of FGF19, the SIRT‐1 inhibitor EX‐527 was applied to C2C12 myotubes. The appropriate concentration (100 µM) of EX‐527 was confirmed for use in subsequent experiments (Figure 3C,D). Then, we found that EX‐527 abrogated the effect of FGF19 on improving the PA‐induced reduction in SIRT‐1 protein levels (Figure 3E,F). Moreover, we identified whether SIRT‐1 was associated with mediating the protective effects of FGF19. This study found that EX‐527 enhanced the protein expression of muscle atrophy markers and aggravated the reduction in myotube diameter induced by PA, thus suppressing the FGF19‐induced improvement in muscle atrophy (Figure 3G‐K). Furthermore, FGF19 did not improve the PA‐induced reduction in p‐IRS‐1 and GLUT‐4 expression with EX‐527 intervention (Figure 3L,M); simultaneously, PA‐induced impaired glucose uptake and lipid droplet accumulation were not mitigated (Figure 3N‐Q). In addition, SIRT‐1 also blocked the beneficial effect of FGF19 on the PGC‐1α and FNDC5 protein expression (Figure 4R‐S). Therefore, activation of SIRT‐1 might be critical for FGF19 to attenuate PA‐induced muscle atrophy, metabolic disturbance and the decline in FNDC‐5/irisin expression.

### 3.4 PGC‐1α might be important in mediating the effects of FGF19 on skeletal muscle

PGC‐1α is a significant downstream effector of AMPK and SIRT‐1 in skeletal muscle. Therefore, we investigated whether PGC‐1α was associated with mediating the protective effects of FGF19 on PA‐induced biological dysfunction in the differentiated C2C12 cells. PGC‐1α knockdown with siRNA transfection significantly suppressed the mRNA and protein expression of PGC‐1α by nearly 60% (Figure 4A‐C). The effect of FGF19 on enhancing PGC‐1α expression was also blocked (Figure 4D,E). Moreover, PGC‐1α‐siRNA treatment increased muscle atrophy marker expression and reduced the myotube diameter; thus, the protective effect of FGF19 on muscle atrophy was dramatically inhibited (Figure 4F,G). In addition, the effect of FGF19 on improving decreased protein expression of p‐IRS‐1, GLUT‐4 and FNDC‐5 induced by PA was markedly abrogated by PGC‐1α‐siRNA (Figure 4H‐K). In conclusion, these results suggested that FGF19 ameliorated PA‐induced impairment of skeletal muscle partially via the PGC‐1α pathway.

### 3.5 Beneficial effects of FGF19 on body weight, muscle mass, grip strength and metabolic parameters in young and aged HFD‐fed mice

**FIGURE 5:**
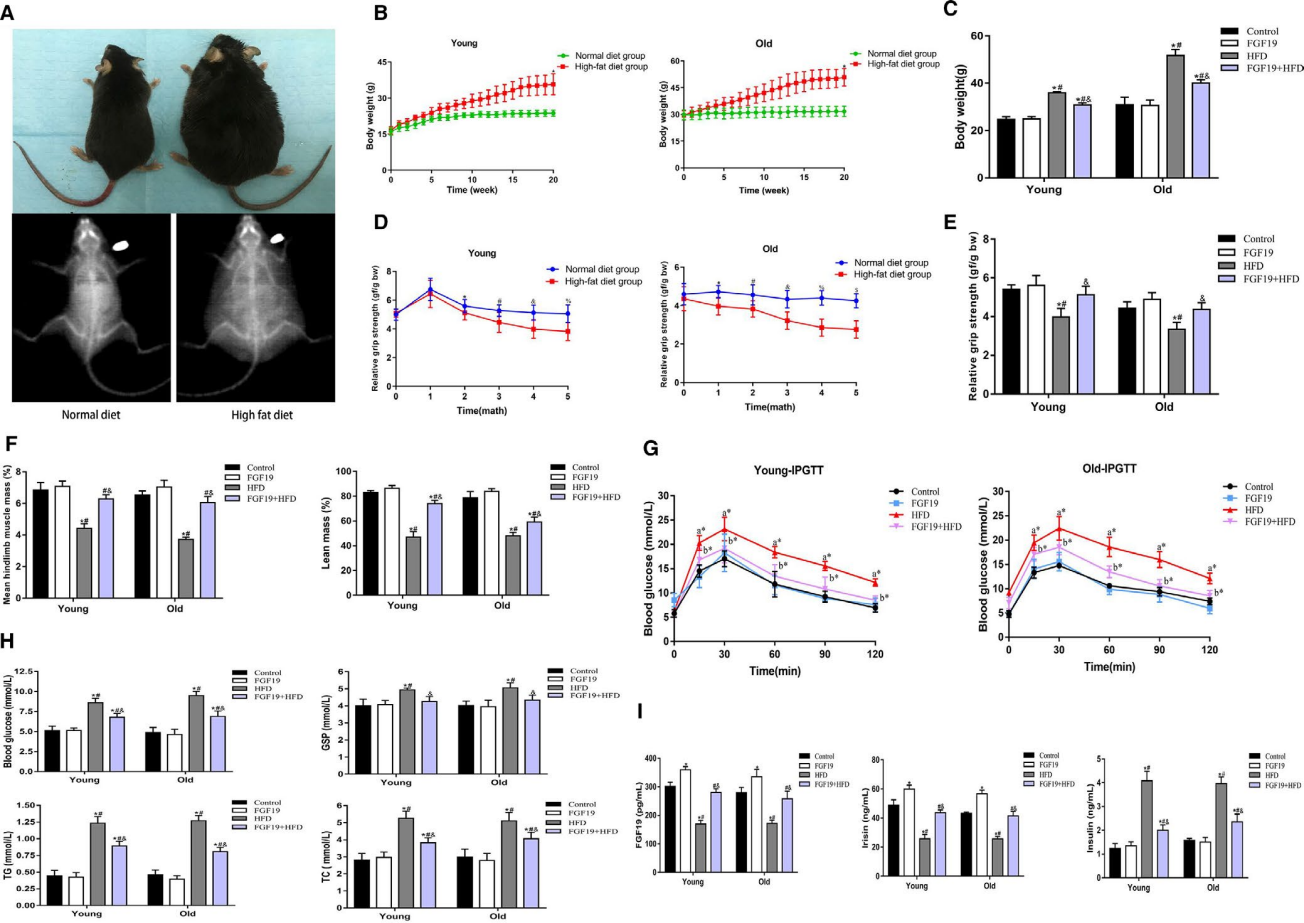
Beneficial effects of FGF19 on body weight, muscle mass, grip strength and metabolic parameters in young and aged HFD‐fed mice. A, Changes in body shape between HFD‐fed and normal diet‐fed mice. B, Dynamic changes in body weight. C, Changes in body weight after FGF19 treatment. D, Dynamic changes in relative grip strength during feeding HFD and normal diet. E, Changes in relative grip strength. F, Changes in mean hindlimb muscle mass and lean mass. G, IPGTT was performed for each group. H, Serum blood glucose, GSP, TG and TC levels were measured in each group. I, Serum FGF19, irisin, and insulin were evaluated by ELISA. All data are presented as the mean ± SEM, *P < 0.05 compared with the control group, ^#^ *P* < 0.05 compared with the FGF19 group, ^&^ *P* < 0.05 compared with the HFD group (n = 6 per group)

To investigate the therapeutic effect of FGF19 on obesity and SO, we examined body weight, lean muscle mass, grip strength and biological parameters in young and old HFD‐fed mice. The body weight of HFD‐fed mice was notably increased by more than 30% compared with normal chow diet‐fed mice, and the old mice showed a more obvious body weight gain than the young mice, while FGF19 treatment dramatically reduced the body weight gain of the obese mice (Figure 5A‐C). In addition, we monitored the grip strength to evaluate the muscle physical function and found that the relative grip strength progressively decreased during ageing and further declined in the aged mice fed an HFD, whereas FGF19 could mitigate HFD‐induced muscle wasting (Figure 5D,E). Furthermore, the decreased percentage of lean mass and mean hindlimb mass in obese mice were significantly mitigated by FGF19 administration to some extent (Figure 5F). Additionally, bodyweight gain in 20‐21‐month‐old aged mice accompanied by a decrease in muscle mass and muscle strength could be considered as a mouse model of SO.

We next investigated the effect of FGF19 on regulating lipid and glucose metabolism. The IPGTT showed that FGF19 treatment ameliorated HFD‐induced impaired glucose tolerance. Moreover, the serological assay identified that the abnormalities in serum fasting blood glucose, GSP and insulin could also be alleviated by FGF19 administration. FGF19 also attenuated HFD‐induced hyperlipidaemia by reducing TG and TC levels. In addition, we found a reduction in FGF19 and irisin levels in the HFD group compared with the control group, whereas FGF19 reversed this effect to some extent (Figure 5G‐I). The above data indicated that FGF19 treatment ameliorated HFD‐induced muscle wasting and dysfunction as well as metabolic disturbance in obese and sarcopenic obese mice.

### 3.6 FGF19 improved HFD‐induced muscle atrophy, lipid accumulation, insulin resistance and abnormalities in FNDC‐5/irisin expression in obese and sarcopenic obese mice

**FIGURE 6:**
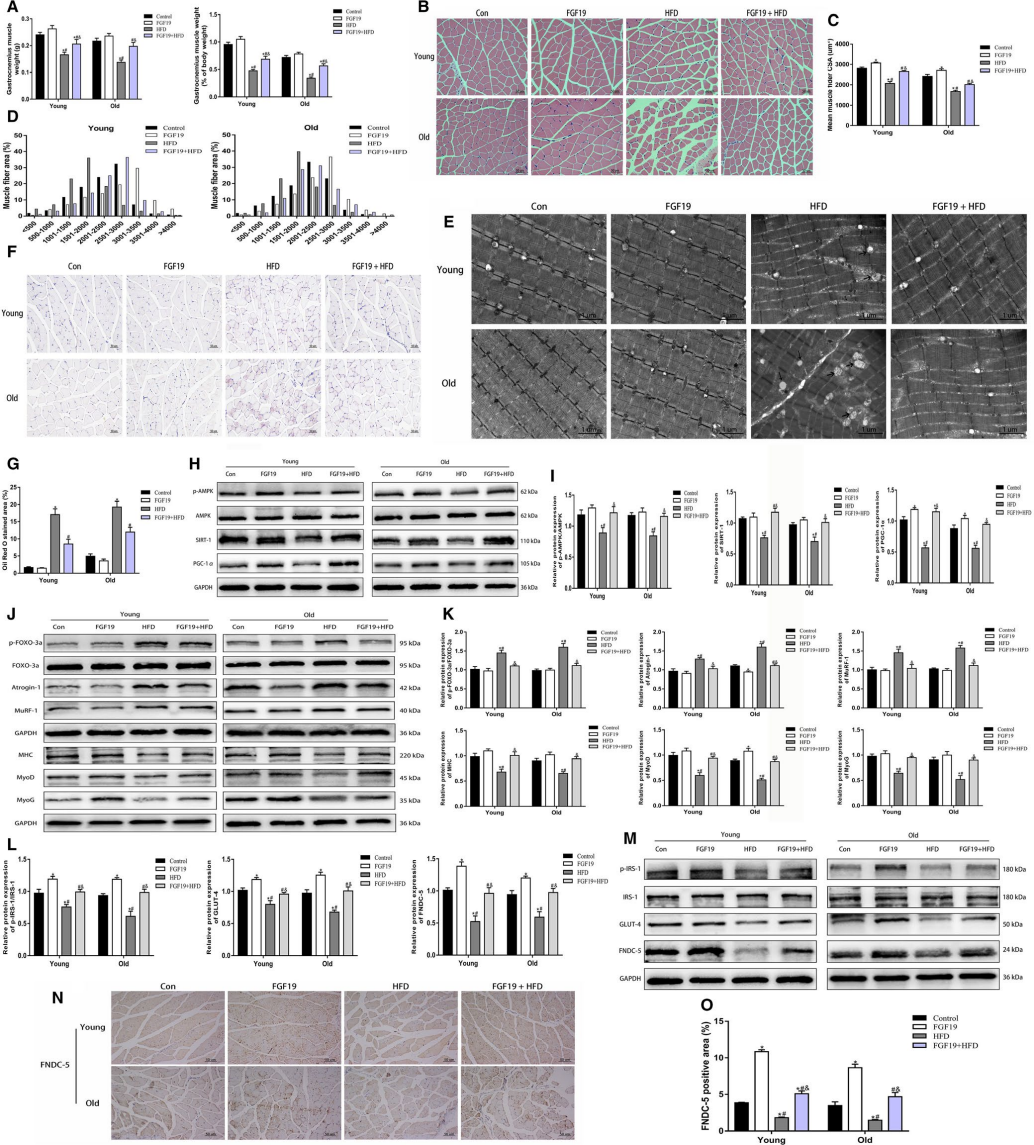
FGF19 improved HFD‐induced muscle atrophy, lipid accumulation, insulin resistance and abnormalities in FNDC‐5/irisin expression in obese and sarcopenic obese mice. A, The weight of gastrocnemius (GAS) muscle and the ratio of GAS muscle weight to body weight (mg/g). B, Representative images of H&E staining for the GAS muscle (scale bar = 50 μm). C, The mean muscle CSA. D, Distribution of muscle fibre CSA in each group. E, Representative TEM images of GAS muscle (scale bar = 2 μm). The thick arrows indicate impaired mitochondria, and the slim arrows indicate lipid droplets. F, G, Representative images of Oil Red O staining of GAS muscle. H, I, Western blot analysis of signalling pathway‐related protein expression (p‐AMPK/AMPK, SIRT‐1, PGC‐1α) in each group. J, K, Western blot analysis of the protein expression of muscle atrophy markers (p‐FOXO‐3a/FOXO‐3a, Atrogin‐1, MuRF‐1) and muscle differentiation markers (MHC, MyoD, MyoG) in each group. L, M, Western blot analysis of glucose uptake (p‐IRS‐1/IRS‐1, GLUT‐4) and FNDC‐5 protein expression in each group. N, O, Representative immunohistochemistry images of FNDC‐5 expression in GAS muscle in each group. All data are presented as the mean ± SEM, *P < 0.05 compared with the control group, ^#^ *P* < 0.05 compared with the FGF19 group, ^&^ *P* < 0.05 compared with the HFD group (n = 3 per group)

We further demonstrated the protective roles of FGF19 in ameliorating HFD‐induced biological dysfunctions of skeletal muscle in obese mice. The results showed that the weight of GAS muscle was reduced during ageing and further decreased in old HFD‐fed mice, but FGF19 supplementation reversed these changes (Figure 6A). H&E staining revealed that FGF19 administration ameliorated the damaged myofibre architecture and decreased the mean fibre CSA in obese mice (Figure 6B‐D). TEM images also revealed that the organization of muscle fibres was damaged in the HFD‐fed mice with disarranged myofilaments and unclear sarcomeres; some mitochondria were swollen with disorganized cristae, some lipid droplets can be visualized. However, this damage induced by HFD could be mitigated by FGF19 (Figure 6E). Furthermore, the Oil Red O staining indicated that lipid droplet infiltration in GAS muscle could also be mitigated (Figure 6F,G). In addition, we identified that HFD‐induced damage to skeletal muscle was more severe in aged mice than in young mice, which might be associated with the risk of ageing.

Next, we further identified whether FGF19 alleviated HFD‐induced biological dysfunction in skeletal muscle by mediating the expression of some related proteins. The results showed that FGF19 treatment abrogated the HFD‐induced reduction in key signalling pathway molecules, including p‐AMPK, SIRT‐1 and PGC‐1α (Figure 6H,I). Then, the results showed that the protein expression of muscle atrophy markers was dramatically up‐regulated, while regulators of glucose metabolism were dramatically reduced in the HFD compared with the control group. However, FGF19 treatment effectively alleviated HFD‐induced muscle atrophy and insulin resistance in skeletal muscle (Figure 6J‐M). In addition, FGF19 treatment promoted FNDC‐5 protein expression and attenuated the HFD‐induced reduction in irisin secretion (Figure 6N,O). Taken together, our results indicated that FGF19 alleviated HFD‐induced muscle atrophy, lipid accumulation, insulin resistance and a reduction in irisin release in the skeletal muscle of obese and sarcopenic obese mice.

### 3.7 FGF19 retarded fat weight gain and enhanced energy expenditure in the BAT of obese and sarcopenic obese mice

**FIGURE 7:**
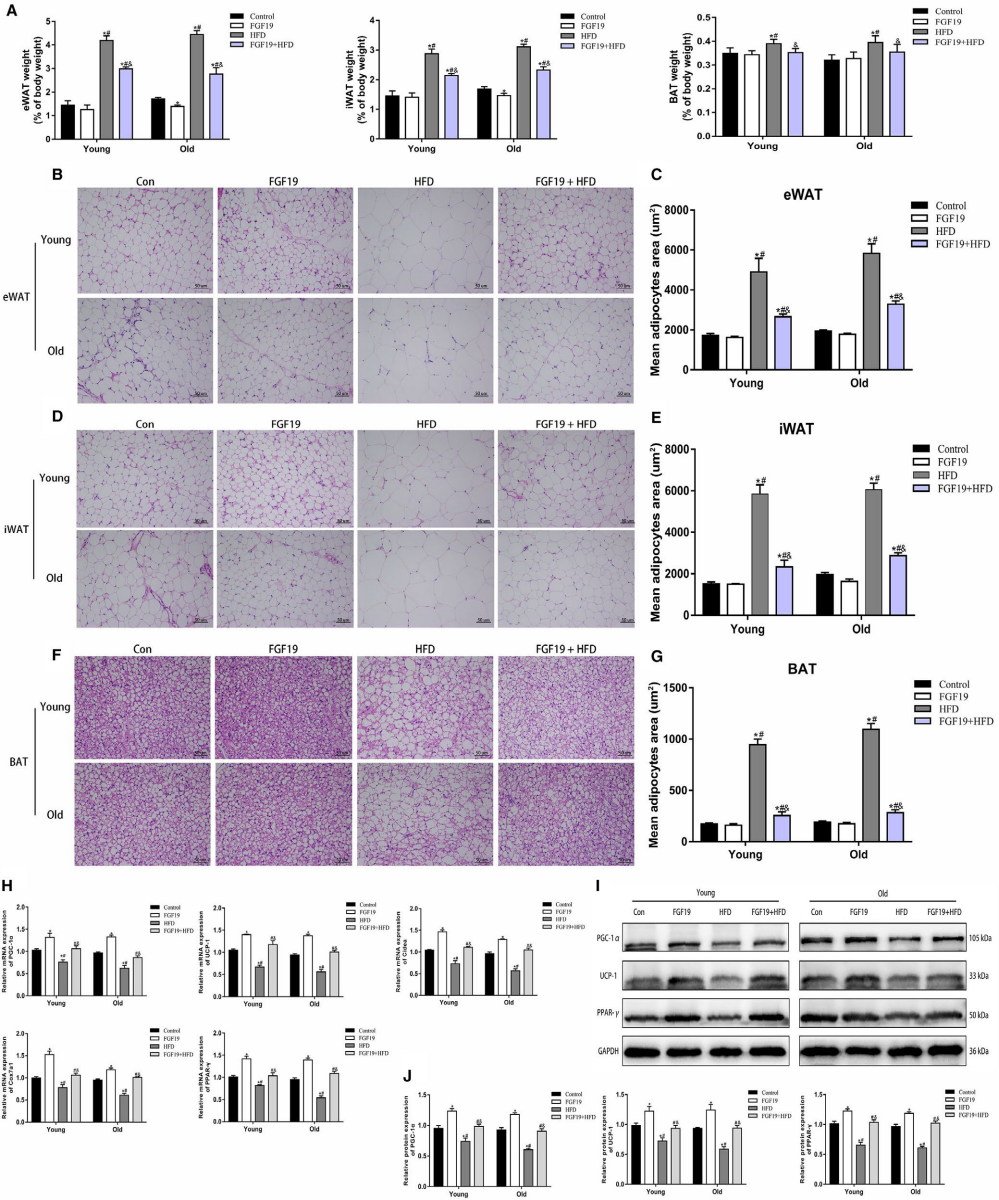
FGF19 retarded fat weight gain and enhanced energy expenditure in the BAT of obese and sarcopenic obese mice. A, The ratio of eWAT, iWAT and BAT weight to body weight. B, C, Representative images of eWAT stained with H&E in each group (scale bar = 50 μm). D, E, Representative images of iWAT stained with H&E in each group (scale bar = 50 μm). F, G, Representative images of BAT stained with H&E in each group (scale bar = 50 μm). H, The mRNA expression of PGC‐1α, UCP‐1, Cidea, Cox7a1 and PPAR‐γ was confirmed by qRT‐PCR. I, J, The protein expression of energy expenditure markers (PGC‐1α, UCP‐1, PPAR‐γ) were identified by western blotting. All data are presented as the mean ± SEM, *P < 0.05 compared with the control group, ^#^ *P* < 0.05 compared with the FGF19 group, ^&^ *P* < 0.05 compared with the HFD group (n = 3 per group)

Some previous studies have identified that obesity can impair energy metabolism in adipose tissues. Previous studies have demonstrated that FGF19 is associated with mediating lipid metabolism. Thus, we identified the regulatory effects of FGF19 on adipose tissues, especially BAT (Figure 7A). The results showed that the ratio of eWAT, iWAT and BAT to body weight was increased in HFD‐fed mice compared with normal diet‐fed mice, and the whitening of BAT in the HFD group became more severe than in the control group. In addition, H&E staining also showed that the adipocytes of eWAT, iWAT and BAT were much larger in HFD‐fed mice than in normal diet‐fed mice (Figure 7B,G). However, FGF19 treatment partially improved these changes in WAT and BAT. Next, we found that the expression levels of some BAT‐associated genes (PGC‐1α, UCP‐1, Cidea, Cox7a1, and PPAR‐γ) and related proteins were significantly reduced in obese mice, while FGF19 could promote the expression of these key molecules (Figure 7H,J). These results indicated that FGF19 might promote energy expenditure and alleviate obesity‐induced abnormalities in lipid metabolism.

## 4 DISCUSSION

Obesity and ageing are associated with biological dysfunctions in skeletal muscle, including lipid infiltration, insulin resistance, inflammation and mitochondrial dysfunction, resulting in the loss of muscle mass and physical dysfunction.^28^, ^29^ It is well‐known that HFD induces obesity and promotes muscle wasting and dysfunction in animals.^30^ Our study revealed a progressive body weight gain and decreased grip strength in HFD‐fed mice. Moreover, these changes in old HFD‐fed mice were more obvious than in young HFD‐fed mice, suggesting the influence of ageing. We constructed mouse models of SO by natural ageing and HFD‐induced obesity occurring simultaneously. According to the evaluation of body weight, fat weight, muscle mass and grip strength, we identified obese and sarcopenic obese mice for use in subsequent experiments. This study found that FGF19 treatment could reduce the final body weight but increase the lean muscle mass and grip strength. Furthermore, the reductions in GAS muscle weight and in the average CSA of GAS muscle were effectively improved. These results suggested that FGF19 treatment significantly alleviated obesity‐induced muscle loss and physical dysfunction.

The balance between protein synthesis and degradation in skeletal muscle is crucial for muscle mass and functions.^31^ When this balance is broken, muscle atrophy generally occurs, which is closely associated with metabolic diseases, including obesity, SO and T2D.^32^ Muscle protein degradation is mainly mediated by two pathways, including the ubiquitin‐proteasome and the autophagy‐lysosomal systems.^33^, ^34^ In this study, we mainly investigated the protein degradation pathway of ubiquitinated protease to indicate the protective effect of FGF19 on obesity‐induced muscle atrophy. Atrogin‐1 and MuRF‐1, as muscle‐specific ubiquitin ligases, play a crucial role in muscle atrophy and are mediated by Forkhead box‐3 (FOXO‐3).^35^, ^36^ In vivo and in vitro, we found that FGF19 could inhibit the protein expression of muscle atrophy markers and enhanced the expression of myogenic differentiation‐related molecules. In addition, our previous research also investigated the effect of autophagy or apoptosis on obesity, T2D or sarcopenia‐induced muscle atrophy by mediating muscle degradation.^37^, ^38^, ^39^ However, the role of FGF19 in mediating autophagy‐lysosome systems might be a novel research target, further experiments are required.

We further hypothesized that FGF19 had protective effects against obesity‐induced muscle atrophy via the AMPK/SIRT‐1/PGC‐1 signalling pathway. Some studies have found that the activation of AMPK by AICAR, an AMPK agonist, restores mouse muscle dysfunction caused by Duchenne muscular dystrophy and reverses inflammation‐ and Ang II‐mediated muscle wasting.^40^, ^41^, ^42^ In addition, activation of AMPK by apigenin and omega‐3 fatty acid‐enriched fish oil can improve PA‐ or HFD‐induced muscle wasting by inhibiting Atrogin‐1 and MuRF‐1.^43^, ^44^ In this study, we found that the AMPK antagonist reversed the beneficial effects of FGF19 on alleviating PA‐induced muscle atrophy. However, some studies have indicated that AMPK can induce protein degradation through regulating FOXO‐3 to promote the ubiquitin‐proteasome pathway and autophagy.^45^, ^46^, ^47^ It has been reported that PGC‐1α has the ability to prevent skeletal muscle atrophy by inhibiting FOXO‐3 and atrophy‐related ubiquitin ligases.^48^, ^49^ Overexpression of PGC‐α is able to prevent the activation of AMPK and reduce the expression of ubiquitinase‐related and autophagic factors.^50^, ^51^ Our data showed that knockdown of PGC‐1α by siRNA transfection can aggravate PA‐induced muscle atrophy and block the protective effect of FGF19. These results indicated that FGF19 might protect skeletal muscle from atrophy by the activation of PGC‐1α. The effect of AMPK/PGC‐1α on inhibiting muscle atrophy might offset the negative effect of AMPK. Additionally, it is well known that the AKT/ERK/mTOR pathway not only promotes muscle protein synthesis but also suppresses FOXO‐3 to prevent muscle protein degradation.^31^ AMPK has the ability to enhance the FOXO‐3 expression via inhibiting the mTOR pathway.^52^ A previous study has reported that FGF19 can promote muscle hypertrophy by activation of the ERK/mTOR pathway, which also might abrogate the effect of AMPK on muscle atrophy.^23^ Moreover, the improvement of mitochondrial dysfunction and lipid/glucose metabolic disturbance indirectly contribute to alleviate muscle atrophy. Therefore, the effect of FGF19 on improving muscle atrophy might comprise comprehensive regulatory mechanisms. These contradictory views also might be associated with the different pathological conditions of muscle atrophy and the different regulatory effects of muscle atrophy‐related signalling pathways.

SIRT‐1 is a critical regulator of muscle metabolism, including muscle growth, differentiation, mitochondrial biogenesis and lipid/glucose metabolism.^53^, ^54^ Hence, activation of SIRT‐1 could be beneficial for improving skeletal muscle diseases.^55^ It has been reported that activation of SIRT‐1 might ameliorate sarcopenia, glucocorticoids and disuse‐induced atrophy.^56^, ^57^ Our study demonstrated that the inhibitory effect of FGF19 on muscle atrophy was abolished by a SIRT‐1 inhibitor, suggesting that FGF19 improved PA‐induced muscle atrophy in part via a SIRT‐1‐dependent pathway.

Skeletal muscle is responsible for regulating lipid and glucose metabolism homeostasis.^58^ Obesity contributes to ectopic fat deposition and IR in skeletal muscle, which impairs muscle biological functions.^59^ Our data showed that FGF19 administration alleviated the hyperglycaemia and impaired glucose tolerance in obese mice. Ectopic lipid accumulation and impaired glucose uptake were also improved by FGF19 in PA‐treated C2C12 myotubes and skeletal muscle of HFD‐fed mice. IRS‐1 (a key molecule in the insulin signalling pathway) and GLUT‐4 (an important glucose transporter) are considered to maintain glucose homeostasis and insulin sensitivity.^60^, ^61^, ^62^ In the present study, we found that the expression of these two molecules was suppressed by PA and HFD, but FGF19 could alleviate this change. Moreover, the metabolic disturbance in obese mice was more severe in aged obese mice than in young obese mice. However, FGF19 improved lipid/ glucose metabolic disturbance in skeletal muscle and even the whole body, suggesting its therapeutic role in obesity and SO.

Muscle glucose and lipid metabolism is considered to be regulated by stimulation of the AMPK/SIRT‐1/PGC‐1α signalling pathway. Some previous studies have suggested that AMPK, SIRT‐1 and PGC‐1α activity can be inhibited by obesity and other metabolic disorders, which impairs lipid and glucose metabolism in skeletal muscle.^26^, ^63^, ^64^ Our data showed that the AMPK, SIRT‐1 and PGC‐1α protein expression levels were significantly decreased in PA‐treated differentiated C2C12 cells and in skeletal muscle of HFD‐fed mice. Furthermore, the inhibition of AMPK, SIRT‐1 and PGC‐1α reversed the beneficial effects of FGF19 on mitigating PA‐ and HFD‐induced lipid droplet accumulation and insulin resistance. Thus, this study indicated that stimulation of the AMPK/SIRT‐1/PGC‐1α signalling pathway might have a significant role in mediating the beneficial effects of FGF19 on lipid and glucose metabolism in skeletal muscle.

Recent studies have suggested a pharmacologic effect of FGF19 on the nervous system to promote insulin sensitivity, energy expenditure and weight loss in adipose tissue.^65^ Thus, we preliminarily identified whether the beneficial effects of FGF19 on obesity were partially associated with mediating the biological functions of adipose tissues, especially BAT. BAT acts as a critical thermogenic organ to promote energy expenditure by activating key regulators of thermogenesis.^66^, ^67^ Obesity profoundly alters BAT morphology and functions, thus affecting whole‐body energy metabolism.^68^ The present study showed that FGF19 treatment markedly reduced the weights of adipose tissues and the size of adipocytes in HFD‐fed mice. The reduction in expression of BAT marker genes and proteins was also improved, thus stimulating BAT thermogenesis. These results indicated that FGF19 might mediate energy metabolism in adipose tissue and ameliorate the obesity‐induced metabolic disturbance. Additionally, irisin promotes energy expenditure in adipose tissues and regulates the biological functions of skeletal muscle.^69^ Thus, FGF19 might also indirectly regulate the effect of adipose tissue by promoting muscle secretion of irisin. Additional studies are needed to investigate the regulatory effects of FGF19 in adipose tissue.

Irisin acts as a crosstalk between skeletal muscle and other organs and tissues, especially adipose tissues.^11^ Since its identification, it has been confirmed to be related to metabolic diseases, including T2D, obesity and NAFLD.^14^ Our study indicated that FNDC‐5 and PGC‐1α expression were dramatically decreased in PA‐treated C2C12 cells and in skeletal muscle of HFD‐fed mice. FGF19 administration reversed this reduction and enhanced circulating irisin levels. Our results are consistent with some previous studies.^70^, ^71^ However, the opposite results have also been found in obese patients and animals.^14^ This controversy might partially be associated with different pathological conditions, intervention methods, secretory tissues and research models.

Irisin is a cleavage product of FNDC5, which is regulated by PGC1‐α.^72^ We found that knockdown of PGC‐1α by siRNA transfection blocked the effect of FGF19 on FNDC‐5 expression. Thus, PGC‐1α might be an essential for FGF19 to regulate the expression of FNDC/irisin. Previous studies have shown that AMPK and SIRT‐1 act as key upstream regulators of PGC‐1α.^72^ In the present study, we identified whether the inhibitory effect of AMPK and SIRT‐1 could abrogate the augmentation of FNDC‐5/irisin expression induced by FGF19 treatment and aggravate PA‐induced abnormalities in FNDC‐5/irisin production. Hence, our study highlights the possible importance of AMPK and SIRT‐1 in the regulation of FGF19 for FNDC‐5/irisin production. At present, a limited number of studies have investigated the relationship between SIRT‐1 and FNDC‐5/irisin in skeletal muscle. This study preliminarily investigated the role of SIRT‐1 in regulating FNDC5 expression in skeletal muscle and provided a novel target for regulating FNDC‐5/irisin production.

This study has investigated the protective role of FGF19 against SO in HFD‐induced sarcopenic obese mice. However, it lacks cellular models of SO to investigate the beneficial effect of FGF19. Previous studies have investigated the underlying mechanism of SO only in animal models. Our research group has used D‐galactose to induce senescence in C2C12 myoblasts.^73^ Whether the combination of PA and D‐galactose could mimic the pathophysiology of SO in vitro is still unclear. After all, the underlying mechanism of SO might be more complicated and not just a simple addition of obesity and ageing.

**FIGURE 8:**
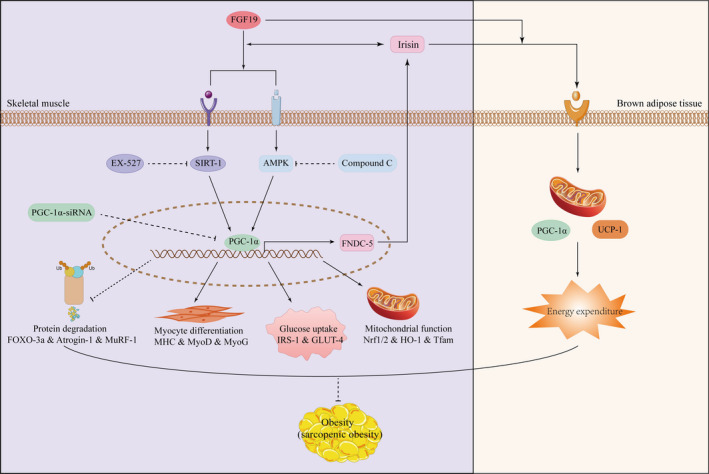
Schematic of the beneficial effects of FGF19 on muscle atrophy, metabolic homeostasis and irisin secretion in skeletal muscle

In conclusion, this study indicated that FGF19 could attenuate muscle atrophy and promote lipid and glucose metabolism and irisin secretion partially through the AMPK/SIRT‐1/PGC‐1α signalling pathway in skeletal muscle (Figure 8). Therefore, FGF19 might become a therapeutic target for improving obesity and SO.

## CONFLICT OF INTEREST

All authors declare no conflicts of interest.

## AUTHOR CONTRIBUTIONS

**Ai Guo:** Conceptualization (lead); Data curation (lead); Formal analysis (lead); Investigation (lead); Methodology (lead); Project administration (equal); Resources (lead); Software (lead); Validation (equal); Visualization (equal); Writing‐original draft (lead); Writing‐review & editing (equal). **Kai Li:** Conceptualization (supporting); Data curation (supporting); Formal analysis (supporting); Investigation (supporting); Methodology (supporting). **Hong‐Chuan Tian:** Conceptualization (supporting); Data curation (supporting); Formal analysis (supporting). **Zhen Fan:** Conceptualization (supporting); Data curation (supporting); Formal analysis (supporting); Investigation (supporting); Methodology (supporting). **Qiu‐Nan Chen:** Data curation (supporting); Formal analysis (supporting). **Yun‐Fei Yang:** Data curation (supporting); Formal analysis (supporting). **Jing Yu:** Data curation (supporting); Methodology (supporting). **Yong‐Xin Wu:** Data curation (supporting); Methodology (supporting). **Qian Xiao:** Conceptualization (supporting); Funding acquisition (lead); Supervision (supporting); Validation (supporting); Visualization (supporting); Writing‐review & editing (supporting).

## Data Availability

The data that support the findings of this study are available from the corresponding author upon reasonable request.
